# Case Report: Surgery combined with extracorporeal membraneoxygenation for acute type A aortic dissection complicated with acute myocardial infarction

**DOI:** 10.3389/fcvm.2025.1463764

**Published:** 2025-01-31

**Authors:** Jianming Xia, Yan Qiu, Shuo Chang, Ying Feng, Heng Zhang, Xiaoqi Wang

**Affiliations:** ^1^ Department of Cardiovascular Surgery, Fuwai Yunnan Hospital, Chinese Academy of Medical Sciences/Affiliated Cardiovascular Hospital of Kunming Medical University, Kunming, China; ^2^ Department of Cardiovascular Surgery, Fuwai Hospital, National Center for Cardiovascular Diseases, Chinese Academy of Medical Sciences and Peking Union Medical College, Beijing, China

**Keywords:** acute myocardial infarction (AMI), acute type A aortic dissection (ATAAD), extracorporeal membrane oxygenation (ECMO), one-stage surgery, malperfusion syndrome

## Abstract

**Background:**

Acute myocardial infarction (AMI) is one of the most serious complications of acute type A aortic dissection (ATAAD) and markedly increases patient mortality. Simultaneous treatment strategies remain controversial. How to improve the treatment of these patients remains a critical challenge for cardiovascular surgeons.

**Case presentation:**

All three patients who experienced chest pain were admitted to emergency department of our hospital. The 12-lead electrocardiogram revealed ST-segment depression, myocardial enzyme levels were significantly elevated. Emergency physicians diagnosed ATAAD with AMI, and emergency surgery was planned in collaboration with the cardiovascular surgery team. One-stage surgery for coronary revascularization and central aortic repair were performed, extracorporeal membrane oxygenation (ECMO) was implanted, ECMO was discontinued when hemodynamics stabilized. The patient ultimately recovered well and was discharged.

**Conclusion:**

For ATAAD combined with right ventricular AMI, one-stage surgery for coronary revascularization and central aortic repair, supported by ECMO as bridge, can be life-saving treatment strategy, the prognosis for all three patients was excellent.

## Introduction

Acute type A aortic dissection (ATAAD) is a highly lethal cardiovascular disease, with the first 24–48 h being a critical time window for mortality (1). Advances in medical technology have led to a gradual decrease in the mortality rate of ATAAD. Notably, based on data from the International Registry of Acute Aortic Dissection (IRAD), Harris et al. (2) found that the 48-hour mortality rate decreased to 23.7% (0.5%/hour) following drug treatment but to 4.4% (0.09%/hour) following surgical treatment. Our strategy for treating ATAAD and reducing mortality emphasizes early diagnosis and immediate surgical treatment. Malperfusion syndrome (MPS) is a severe complication of ATAAD, characterized by high mortality and poor clinical outcomes despite advancements in diagnosis and treatment strategies (3). ATAAD combined with myocardial infarction (AMI) represents one of the most serious forms of MPS (4). Currently, no treatment guidelines have been established for ATAAD combined with AMI, and treatment strategies remain controversial, with successful case outcomes primarily documented in case reports. In recent years, we have successfully treated three cases of ATAAD complicated with AMI using surgery and extracorporeal membrane oxygenation **(**ECMO) in combination, as shown in Table 1. This summary aims to enhance our understanding and management of this complex condition.

**Table 1:** Baseline characteristics of ATAAD complicated with AMI.

	Case 1	Case 2	Case 3
Age (years)	44	41	58
Sex (Male/female)	Male	Male	Male
Height (cm)	168	170	170
Weight (Kg)	88.5	49	68
Classification of acute myocardial infarction	Non- ST-segment elevation myocardial infarction (NSTEMI)	NSTEMI	NSTEMI
Aortic sinus diameter (mm)	57	50	57
Ascending aortic diameter (mm)	50	50	41
Left ventricular end diastolic diameter (mm)	40	51	66
Ejection fraction (%)	59	58	42
Platelet (×10^9^)	152	115	160
Creatinine (µmol/L)	107	66.3	122
Disease onset-operation time	27	65	28
Classification of Neri	B	B	C
Drainage fluid on first day after surgery (ml)	750	870	910
Input red blood cells (u)	11.5	12u	10u
Input plasma (ml)	2,200	1,400	400
Input platelets (u)	2	4u	2u
ECMO time (h)	83	97	106
IABP time (h)		168	
CRRT time (h)			504
Ventilator time(h)	136	213	221
ICU time (h)	205	430	677
Hospital stays (h)	589	982	677

## Case reports

### Case 1

**Figure 1:**
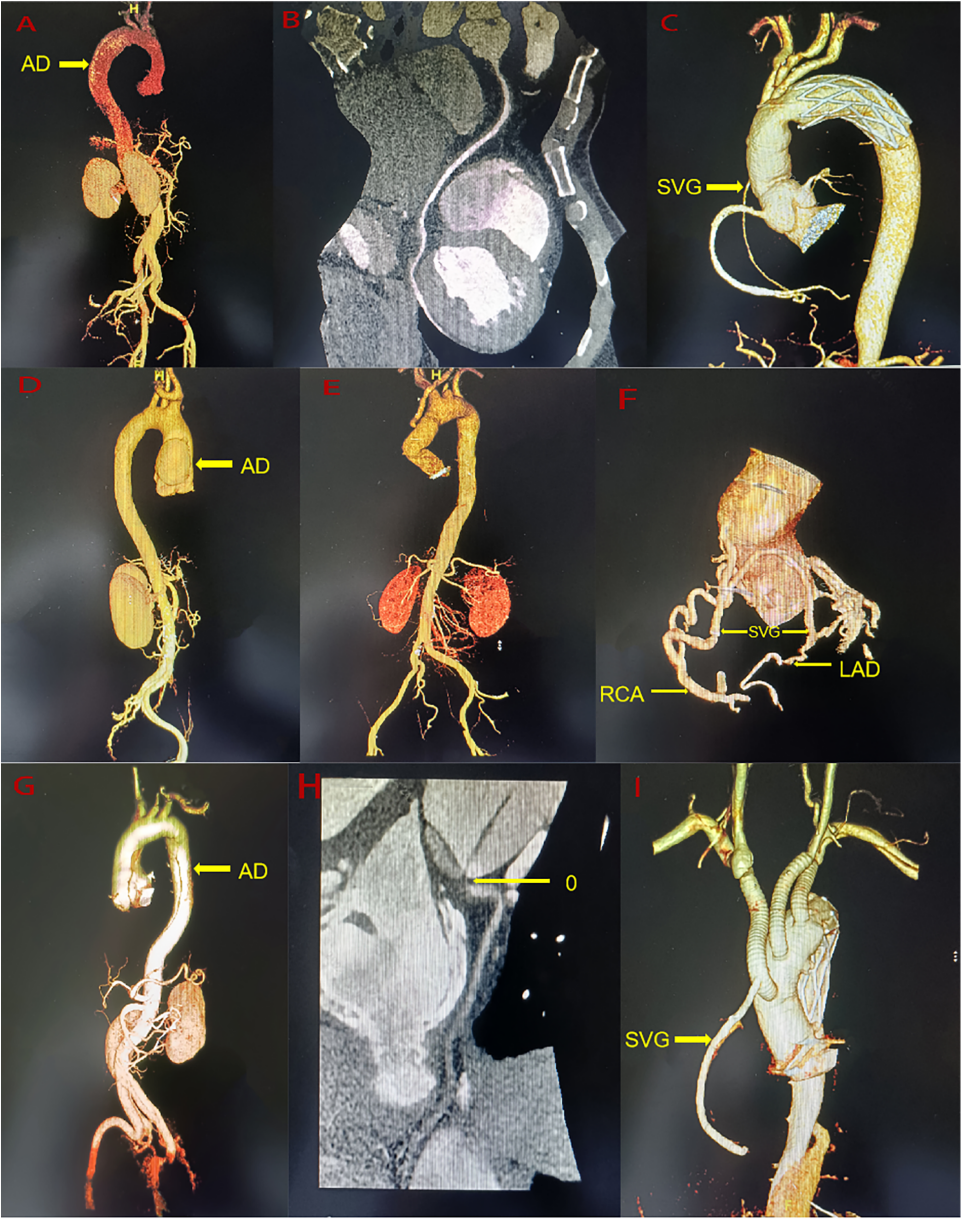
**(A)** A pre-operative the entire aorta CTA of case 1. **(B)** A pre-operative the right coronary artery CTA of Case 1. **(C)** A post-operative the entire aorta CTA of Case 1. **(D)** A pre-operative the entire aorta CTA of Case 2. **(E)** A post-operative the entire aorta CTA of Case 2. **(F)** A post-operative the coronary artery CTA of Case 2. **(G)** A pre-operative the entire aorta CTA of Case 3. **(H)** A pre-operative the right coronary artery CTA of Case 3. **(I)** A post-operative the entire aorta CTA of Case 3. AD, aortic dissection; RAC, right coronary artery; LAD, anterior descending branch; SVG, saphenous vein graft.

A 44-year-old man (height 168 cm, weight 88.5 kg) was admitted to the emergency department of our hospital with chest pain persisting for 8 h. He had a history of hypertension but did not take medication regularly. His vital signs upon admission included a right arm blood pressure of 138/77 mmHg, a pulse of 70 beats/min, a respiratory rate of 18 breaths/min, and an oxygen saturation (SpO2) of 97%. A 12-lead electrocardiogram revealed ST-segment depression in the II, III, and aVF leads. Myocardial enzyme levels were significantly elevated, including CK-MB at 182 ng/ml (0–6 ng/ml) and troponin at 24.54 ng/ml (0–0.08 ng/ml). A pre-operative computed tomography angiogram (CTA) of the entire aorta, coronary artery and supra-aortic vessels revealed ATAAD with a rupture located in the ascending aorta (Figure 1A–B). The dissection flap extended from the ascending aorta to bilateral common iliac artery, right external iliac artery and left internal iliac artery. Celiac trunk artery, superior mesenteric artery, bilateral renal artery and most of intercostal arteries originate from the true lumen, while the inferior mesenteric artery originate from the false lumen. Eccentric thickening of the wall of the proximal segment of the right coronary artery and the proximal segment of the left ventricular posterior branch was observed. A small aneurysm was noted in the M2 segment of the left middle cerebral artery, and the lumen of the right anterior cerebral artery appeared slender. Pre-operative echocardiography revealed coordinated movement of the left and right ventricles with normal contraction amplitude and a left ventricular ejection fraction (LVEF) of 59%. Emergency physicians diagnosed ATAAD with AMI, and emergency surgery was planned in collaboration with the cardiovascular surgery team. For the surgery performed via median sternotomy, cardiopulmonary bypass (CPB) was established through the axillary artery, femoral artery, and right atrium. The ascending aorta was clamped proximal to the brachiocephalic artery and opened to inspect the coronary ostial lesions. A cold blood cardioplegic solution was perfused through the coronary ostium, and no intimal tear was found. The heart was covered with ice. The dissected aorta was resected, intima and media of the aorta were resected, the false lumen blood clots were cleared, and a large epicardial hematoma on the right ventricular surface was explored. Sequential coronary artery bypass grafting (CABG) was performed using a saphenous vein graft from the left ventricular posterior branch (PL) to the posterior descending branch (PDA). Proximal aortic reconstruction was performed using the adventitial inversion technique. Circulatory arrest was initiated at a bladder temperature of 28℃ with bilateral-anterograde cerebral perfusion. A 30-mm self-expandable elephant trunk stent graft was inserted into the true lumen of the descending aorta, and aortic arch reconstruction was performed using a 30-mm four-branched arch graft. When the heart resumed beating and body temperature normalized, intraoperative transesophageal echocardiography revealed decreased left heart function with an LVEF of 32%, the patients have evidence of cardiogenic shock (systemic systolic pressure <90, urine output <30 ml/hour, lactate >2), necessitating the initiation of ECMO through the axillary artery and femoral vein (rotation speed: 2,500 R/min, flow rate: 3.360 L/min). The right coronary saphenous vein graft flow was 58 ml/min, and the pulsatility index was 2.0 by transit time flow measurement (TTFM). The CPB duration was 337 min, aortic cross-clamping time was 153 min, and the circulatory arrest time was 19 min. ECMO was discontinued on post-operative day 3, and the patient was extubated on day 7. The patient spent 18 days in intensive care and 24 days in the hospital. Echocardiography at discharge revealed hypokinesis of the middle and the lower segments of the right ventricle and lower posterior wall of the left ventricle, with an LVEF of 55%. Ultrasound at discharge revealed venous thrombosis in the bilateral lower limbs and right upper limbs. The CTA revealed bilateral scattered pulmonary embolism with an unobstructed artificial vessel and a well-dilated elephant trunk stent without internal leakage. The false lumens distal to the stent had thrombosed. At 2 years and 6 months of follow-up, the patient exhibited normal heart function and could perform normal daily activities. Recent echocardiography revealed normal left and right ventricular wall movements, with an LVEF of 63%. Recent CTA revealed an unobstructed great saphenous vein graft, occlusion of the left ventricular posterior branch, unobstructed artificial vessels, a well-attached elephant trunk stent to the aortic wall, thrombosis and occlusion of the false lumen (Figure 1C).

### Case 2

A 41-year-old man (height 170 cm, weight 49 kg) was admitted to the emergency department of our hospital with persistent chest pain for 2 days. He had no prior medical history. His vital signs upon admission included a right arm blood pressure of 122/75 mmHg, a pulse of 99 beats/min, a respiratory rate of 17 breaths/min, and a SpO2 of 97%. A 12-lead electrocardiogram revealed ST-segment depression in leads V4-V6. Laboratory tests showed elevated troponin at 2.54 ng/ml (0–0.08 ng/ml) and CK-MB at 6.76 ng/ml (0–6 ng/ml). Pre-operative CTA of the entire aorta, coronary artery and supra-aortic vessels revealed ATAAD, with the entry located in the ascending aorta (Figure 1D). The dissection was limited from the aortic sinus to ascending aorta, with a penetrating ulcer in the initial segment of the left carotid artery, severe stenosis of celiac trunk artery, but no significant stenosis in the coronary arteries. Pre-operative echocardiography revealed mild-to-moderate aortic valve regurgitation and normal ventricular wall movement, with an LVEF of 58%. Emergency physicians diagnosed type A AD with AMI, with surgery arranged after discussion with the Cardiovascular Surgery team. The surgery was performed via median sternotomy, which included establishing CPB through the axillary artery and right atrium. The ascending aorta was clamped proximal to the brachiocephalic artery and opened to inspect the coronary ostial lesions. Cold del Nido cardioplegic solution was administered through the coronary ostium with no intimal tear observed. The heart was covered in ice, and the intima and media of the aortic dissection were resected. The right-non-junction of the aortic valve was repaired with a 5-0 suture. Aortic sinus reconstruction was conducted using the adventitial inversion technique and bovine pericardium. Replacement of the ascending aorta was performed using a 30-mm straight vessel. The left carotid artery was clamped, and the penetrating ulcer was resected and replaced with an 8-mm straight graft. After the heart resumed beating, the aortic sinus dissection progressed, causing a hematoma that compressed the left and right coronary arteries. Esophageal ultrasound revealed obstruction on coronary blood flow and moderate aortic valve regurgitation. The ascending aorta was reclamped, and the anastomotic site was opened. Cold del Nido cardioplegic solution was directly perfused anterogradely from the coronary ostial. A thorough resection of the sinus dissection tissue was performed, the aortic valve was removed, and Bentall surgery was conducted. After the heart resumed beating, the movement of the right and left ventricular anterior walls was weakened. CABG was performed using a saphenous vein for the left anterior descending artery (LAD) and right coronary artery (RCA) during CPB. Intraoperative transesophageal echocardiography revealed decreased left and right heart function with an LVEF of 30%. The patients have evidence of cardiogenic shock (systemic systolic pressure <90, urine output <30 ml/hour) until after an intra-aortic balloon pump (IABP) was inserted, necessitating the initiation of ECMO through the femoral artery and femoral vein to unload the heart and restore cardiac function (rotation speed: 2,100 R/min, flow rate: 3.0 L/min). The RCA-saphenous vein graft flow was 72 ml/min, and the pulsatility index was 2.1 by TTFM. The LAD-saphenous vein graft flow was 58 ml/min, and the pulsatility index was 2.0 by TTFM. The CPB duration was 768 min, and the aortic cross-clamping time was 182 min. ECMO was discontinued on post-operative day 4. IABP was removed on post-operative day 7. The patient was extubated after 8 days and 21 h. The patient spent 17 days in intensive care and 40 days in the hospital. Echocardiography at discharge revealed normal left and right ventricular wall movement, with an LVEF of 60%. A CTA at discharge revealed an unobstructed ascending aorta and an artificial left carotid artery blood vessel (Figure 1E–F). At the 2 years and 4 -month follow-ups, the patient exhibited normal heart function and could perform normal daily activities. Recent echocardiography revealed normal left and right ventricular wall movement, with an LVEF of 58%. Recently, CTA revealed an unobstructed saphenous vein vessel, 50%–70% stenosis in the proximal right coronary artery, irregular and mild stenosis in the left main artery, unobstructed artificial blood vessels in the ascending aorta, and an artificial left carotid artery blood vessel.

### Case 3

A 58-year-old man (height 170 cm, weight 68 kg) was admitted to emergency department of our hospital after experiencing chest pain for 22 h. His vital signs upon admission included right arm blood pressure of 87/51 mmHg, a pulse rate of 89 beats/min, a respiratory rate of 17 breaths/min, and a SpO2 of 98%. A 12-lead electrocardiogram revealed ST-segment depression in the I and aVL leads and pathological Q waves in II, III, and aVF leads. Myocardial enzyme levels were significantly elevated, with CK-MB at 142 ng/ml (0–5.85 ng/ml) and troponin exceeding 3 ng/ml (0–0.018 ng/ml). The Pre-operative CTA of the entire aorta, coronary artery and supra-aortic vessels showed ATAAD with the rupture located in the ascending aorta, dissection flap extending from the root of the aorta to both internal and external iliac artery, aortic sinus aneurysm, and occlusion of the right coronary ostial (Figure 1G–H). Superior mesenteric artery, right renal artery, the inferior mesenteric artery and most of intercostal arteries originate from the true lumen, while celiac trunk artery and left renal artery originate from the false lumen. Pre-operative echocardiography revealed severe aortic regurgitation, with an LVEF of 42%. Emergency physicians diagnosed ATAAD with AMI, with emergency surgery arranged after discussion with the Cardiovascular Surgery team. The surgery performed in median sternotomy, involved establishing CPB through femoral artery and right atrium. The ascending aorta was clamped proximal to the brachiocephalic artery and opened to inspect the coronary ostial lesions. The left coronary ostial was normal, but the right coronary ostial displayed an intima tear located beside the right coronary artery ostium but not inside the right coronary artery ostium. Cold blood cardioplegic solution was perfused through the coronary ostium, and the heart was covered with ice. CABG was performed using a saphenous vein for the RCA, and the right coronary artery ostial was closed with retrograde cold blood cardioplegic solution through the saphenous vein. The aortic valve was resected, and Bentall surgery was performed. Bilateral cerebral perfusion was established through the right innominate artery and left carotid artery. Circulation arrest was induced when the bladder temperature dropped to 28 ℃, combination with bilateral anterograde cerebral perfusion. A 24-mm self-expandable elephant trunk stent graft was inserted into the true lumen of the descending aorta, and aortic arch reconstruction was performed using a 26-mm four-branched arch graft. When the heart resumed beating and body temperature returned to normal, Intraoperative transesophageal echocardiography revealed decreased left and right heart function with an LVEF of 35%, the patients have evidence of cardiogenic shock (systemic systolic pressure <90, urine output <30 ml/hour, lactate >2), necessitating the initiation of ECMO through the femoral artery and femoral vein (rotation speed: 2 200 R/min, flow rate: 3.2 L/min). The RCA-saphenous vein graft flow was 47 ml/min, the pulsatility index was 4.2 by TTFM. After returning to surgical intensive care unit (SICU), the patient exhibited no urine output and high lactate levels, requiring continuous renal replacement therapy (CRRT). The CPB duration was 307 min, aortic cross-clamping time was 184 min, and circulatory arrest time was 21 min. ECMO was discontinued on post-operative day 4 and the patient was extubated on day 9. The patient remained in the SICU for 28 days, before being transferred to the rehabilitation department. Echocardiography at discharge revealed a diffuse decline in left ventricular wall contraction and normal right ventricular wall movement, with an LVEF of 43%. Ultrasounds at discharge revealed right lower limb venous thrombosis. The CTA at discharge indicated unobstructed artificial and saphenous vein grafts, a well-expanded elephant trunk stent with no internal leakage, thrombosed false lumens distal to the stent, and a right upper lobe pulmonary embolism (Figure 1I). At 3-month follow-up, the patient showed a diffuse decline in heart function but was able to engage in normal daily activities. Recent echocardiography revealed a diffuse reduction in left ventricular wall contraction, while right ventricular wall movement remained normal, with an LVEF of 48%.

## Discussion

The primary causes of mortality in ATAAD are aortic rupture and malperfusion (5). The objective of the surgery intervention is to excise the dissection in the ascending aorta and aortic arch to prevent aortic rupture and restore organ perfusion, thereby preventing necrosis. For uncomplicated ATAAD, timely central aortic repair is widely recognized as the most effective and safest treatment option. However, the occurrence of MPS markedly increases patient mortality and significantly worsens the prognosis (6, 7). Addressing how to improve the treatment of these patients remains a critical challenge for cardiovascular surgeons.

The incidence of ATAAD combined with AMI ranges from 5 to 7.1% (2, 8), and the associated mortality rate is extremely high (9). Currently, there is no large-scale clinical data available on the treatment outcomes for this condition, with surgical treatment only documented in a few cases (10). In the cases reported here, we optimized the surgical treatment strategy for ATAAD combined with AMI. Following coronary revascularization and central aortic repair, ECMO was employed as a “bridge-to-recovery” to help stabilize hemodynamics. The prognosis for all three patients was excellent. To date, only two relevant reports using this treatment approach have been published, as shown in Table 2 (11, 12).

**Table 2:** Summary of patients with ATAAD complicated with AMI treated by surgery combined with ECMO.

Reference	11	12	Case 1	Case 2	Case 3
Age (years)	36	42	44	41	58
Sex (Male/female)	Female	Male	Male	Male	Male
Diagnosis	ATAAD combined with AMI	ATAAD combined with AMI	ATAAD combined with AMI	ATAAD combined with AMI	ATAAD combined with AMI
Disease onset- operation time (hours)	144	unrecorded	27	65	28
Operation	Ascending aorta and total aortic arch replacement combined with elephant trunk stent implantation	Ascending aorta and total aortic arch replacement combined with elephant trunk stent implantation	Aortic sinus reconstruction + ascending aorta and total aortic arch replacement combined with elephant trunk stent implantation + CABG	Bentall + CABG	Bentall + total arch replacement and elephant trunk stent implantation + CABG
Coronary processing	True lumen of RCA not found; CABG halted	RCA recovery patency by PCI	Sequential CABG was performed from PL and PDA using saphenous vein graft	CABG was performed using saphenous vein for LAD and RCA	CABG was performed using saphenous vein for RCA
ECMO time(hours)	312	120	72	96	96
Normal daily activities	Yes	Yes	Yes	Yes	Yes
Follow-up time(months)	3	6	30	28	3

AMI, due to the propagation of an aortic dissection into the coronary arterial wall or compression of the coronary arteries by a hematoma, is a critical and often fatal condition (13). The pathophysiological process involves bulging of the dissected false lumen at the branch orifice, leading to distal thrombosis, intimal detachment, and further extension of the dissection into the branch (14). In contrast to stable aortic dissection, AMI represents an immediate, life-threatening complication. The optimal treatment strategy remains controversial, with ongoing debate between one-stage and staged surgery. One-stage surgery can simultaneously address both aortic rupture and myocardial ischemia but is associated with a high surgical mortality rate (9). To mitigate surgical risks, a staged approach has been proposed, starting with percutaneous coronary intervention (PCI) bridging to restore coronary blood flow and heart function, followed by center aortic repair. However, this approach risks aortic rupture before definitive aortic repair (15, 16). Cardiovascular surgeons are thus confronted with difficult choices and challenges. Considering the specific characteristics of the lesions in the three patients, we carefully considered and optimized our treatment strategy.

We opted not to perform PCI for the three patients, instead choosing a one-stage surgical approach for coronary revascularization and central aortic repair. This was decided based on the following rationale: (1) Performing PCI in cases of ATAAD carries the risk of aortic rupture. Not all coronary artery involvements caused by ATAAD can be corrected with PCI; some require surgical repair of both the aorta and coronary arteries or simultaneous CABG. (2) Performing PCI requires the use of anticoagulants, increasing the risk of bleeding during surgery. There is also a significant risk of stent thrombosis post-surgery, often requiring CABG, thereby complicating the overall treatment. (3) The three patients, presenting with NSTEMI demonstrated relatively stable circulatory conditions, categorizing them as a low-risk population of acute ischemic events. (4) The patients primarily experienced right ventricular myocardial infarction. The right ventricle exhibits remarkable resilience to ischemic injury and a strong capacity for recovery even after prolonged occlusion. Consequently, the term “right ventricular infarction” is somewhat misleading, as an acutely ischemic right ventricle remains largely viable and robust (17). While right ventricular function can improve spontaneously without reperfusion, recovery can be slow and is often associated with significant in-hospital morbidity and mortality. Reperfusion accelerates the recovery of right ventricular function and improves overall clinical outcomes and survival rates. Additionally, using ECMO as a bridge can help reduce mortality and complications. Wang et al. (11) reported a case of complete right coronary artery occlusion associated with type A aortic dissection, where CABG was not performed, and right ventricular function gradually recovered postoperatively with ECMO support. Similarly, the three patients discussed experienced full recovery of their right heart function following surgery.

Neri et al. (18) introduced the famous Neri classification system based on the extension of the dissection. This system is both simple and practical for making decisions during surgery. According to this classification, the coronary ostia can be directly repaired for Neri A and several Neri B types, while coronary artery bypass grafting can be performed for certain Neri B types and Neri C types (19). Given preoperative myocardial injury and the potential for intraoperative cardiac arrest, myocardial protection during surgery is crucial. Therefore, exploration of coronary ostia lesions before cardioplegic perfusion is necessary to ensure effective myocardial protection during surgery (20). The cardioplegic solution is then perfused through the coronary ostium if no intimal tear is found. After performing CABG, retrograde perfusion is completed via a saphenous vein graft. If no intimal tear is identified, cardioplegic solution can be safely delivered through the coronary ostium. Following CABG, retrograde perfusion via a saphenous vein graft is completed to further optimize myocardial perfusion. In cases where anterograde perfusion through the coronary ostium is not feasible, direct perfusion into the coronary artery or retrograde delivery via the coronary sinus serve as effective alternatives. While coronary CTA is the primary imaging modality for preoperative coronary evaluation, its accuracy is lower than that of coronary angiography, so results should be interpreted with caution. During surgery, meticulous exploration of the coronary arteries is necessary to determine the need and precise location for CABG. In case 2, due to an inaccurate coronary CTA assessment and weakened heart function after reperfusion, an unplanned CABG was required.

Previous research has shown no difference in mortality between early CABG (within 72 h) and late CABG (beyond 72 h), with a similar incidence of major adverse cardiac and cerebrovascular events (MACCE) at 6-month follow-up among NSTEMI patients (21). IABP is a common device used to increase coronary blood flow. However, the IABP acute myocardial infarction complicated with cardiogenic shock, the IABP-SHOCK trial demonstrated that IABP cannot reduce all-cause mortality in AMI complicated by cardiogenic shock (22). As a result, current European guidelines have downgraded the recommendation for IABP use from class I C to class III B (23). Recently, the use of alternative devices such as Impella, TandemHeart, and ECMO has increased significantly. ECMO, which reduces cardiac preload and oxygen consumption while providing circulatory and respiratory support, has become our preferred and most critical assistive device for successfully weaning patients off CPB. In case 2, where there was no dissection in the descending aorta, both IABP and ECMO were used. In contrast, in Cases 1 and 3, where descending aortic dissection was present, only ECMO was employed.

We use heparin anticoagulant, an ACT range of 180–220s and an APTT range of 60–80s have been suggested for ECMO. Patients with type A dissection experience significant disruption in their coagulation function, which is further exacerbated by surgical trauma and the administration of large volumes of blood products. Consequently, post-surgery coagulation is often severely impaired. In addition, prolonged bed rest during recovery increases the risk of thrombosis. In the cases reported here, two developed pulmonary embolism, and two developed deep vein thrombosis in their limbs.

All three patients showed favorable recovery post-surgery, with follow-up periods ranging from 3 months to 2 years and 6 months. Postoperative evaluations indicated that both artificial vessels and saphenous vein grafts remained unobstructed, and right heart function had normalized, enabling patients to resume normal daily activities.

In conclusion, ATAAD combined with AMI is a rare and complex condition with no established international treatment guidelines. Surgical intervention is highly challenging, but with an experienced cardiovascular diagnosis and treatment team, one-stage surgery for coronary revascularization and central aortic repair, supported by ECMO as a bridge, can be life-saving.

## Data Availability

The original contributions presented in the study are included in the article/Supplementary Material, further inquiries can be directed to the corresponding author.
